# Proposal of Lactobacillus amylovorus subsp. animalis subsp. nov. and an emended description of Lactobacillus amylovorus

**DOI:** 10.1099/ijsem.0.006517

**Published:** 2024-09-12

**Authors:** Kenji Yamane, Yasuhiro Tanizawa, Hisami Kobayashi, Tomomi Kamizono, Yoichiro Kojima, Hiroki Takagi, Masanori Tohno

**Affiliations:** 1 Innovative Animal Production System, University of Tsukuba, 1-1-1 Tennodai, Tsukuba, Ibaraki 305-8571, Japan; 2 Nihon Shokuhin Kako Co. LTD, 30, Tajima, Fuji, Shizuoka 417-8530, Japan; 3 Department of Informatics, National Institute of Genetics, Mishima, Shizuoka 411-8540, Japan; 4 Institute of Livestock and Grassland Science, National Agriculture and Food Research Organization, Nasushiobara, Tochigi 329-2793, Japan; 5 Research Center of Genetic Resources, National Agriculture and Food Research Organization, Tsukuba, Ibaraki 305-8602, Japan

**Keywords:** *Bacillota*, lactic acid bacteria, *Lactobacillus amylovorus* subsp*. animalis*

## Abstract

A corrigendum of this article has been published full details can be found at 10.1099/ijsem.0.006564

Seven novel lactic acid bacterial strains (BF125^T^, BF186, TKL145, YK3, YK6, YK10 and NSK) were isolated from the fresh faeces of Japanese black beef cattle and weanling piglets, spent mushroom substrates, or steeping water of a corn starch production plant. These strains are rod-shaped, Gram-stain-positive, non-motile, non-spore-forming, catalase-negative, cytochrome oxidase-negative, facultatively anaerobic, and homofermentative. Strain BF125^T^ did not produce any gas from glucose; both d- and l-lactate were produced as end-products of glucose (D/L, 40 : 60). Growth occurred at 30–45 °C (optimum, 37 °C), pH 5.0–8.0 (optimum, pH 6.0), and with NaCl concentration of 1.0–3.0% (w/v). The G+C content of genomic DNA of strain BF125^T^ was 37.8 mol% (whole-genome analysis). The major fatty acids were C_16 : 0_, C_18 : 1_ ω9*c*, C_19_ cyclopropane 9, 10*,* and summed feature 10. The 16S rRNA gene in strain BF125^T^ showed high similarity to that of the type strain of *Lactobacillus amylovorus* (99.93%), and the other isolates were also identified as *L. amylovorus* based on these similarities. A phylogenetic tree based on the core genomes of *L. amylovorus* strains (*n*=54), including the seven isolates, showed that they could be divided into two clusters. Strains YK3, YK6, YK10, and NSK were in the first cluster, along with the type strain DSM 20531^T^, while the second cluster included isolates BF125^T^, BF186, TKL145, and other strains isolated from various animal origins. Phenotypic differences in fermentability were observed for lactose, salicin, and gentiobiose between these two groups. The intergroup digital DNA–DNA hybridization values (72.9–78.6%) and intergroup average nucleotide identity values (95.64–96.92%) were comparable to values calculated using datasets of other valid subspecies of the genus (ex-) *Lactobacillus*. In light of the physiological, genotypic, and phylogenetic evidence, we propose a novel subspecies of *L. amylovorus*, named *Lactobacillus amylovorus* subsp. *animalis* subsp. nov. (type strain BF125^T^=MAFF 212522^T^=DSM 115528^T^). Our findings also led to the automatic creation of *Lactobacillus amylovorus* subsp. *amylovorus* subsp. nov. and an emended description of the species *L. amylovorus*.

## Introduction

Lactic acid bacteria exist in various environments, including human and animal bodies, and have long been used for the production of fermented foods, such as yoghurt, cheese, pickles, and sake. Recently, the functions of lactic acid bacteria (intestinal regulation, immunomodulation, prevention of arteriosclerosis, and antitumor action) were scientifically proven, and their effects on human health have gained increasing attention [1].

*Lactobacillus* is the main genus of lactic acid bacteria and is Gram-stain-positive, rod-shaped, non-spore-forming, and facultatively anaerobic [2]. Recently, many genome comparisons of *Lactobacillus* have been reported, owing to advances in next-generation sequencing technology. In 2020, Zheng *et al*. conducted a genomic-level reclassification of all *Lactobacillus* species previously classified in this genus [3]. The analyses included average nucleotide identity (ANI), average amino acid identity, and core genome phylogeny. Based on these analyses, *Lactobacillus* was reclassified into 25 genera and 23 new genera were proposed. At the time of writing of this article, the genus *Lactobacillus* comprised 44 validly named species (www.bacterio.net; List of Prokaryotic names with Standing in Nomenclature) [4].

*Lactobacillus amylovorus* belongs to the genus *Lactobacillus*, and its type strain has been isolated from cattle waste-corn fermentations (CCFs) [5]. In this plant-related environment, the final microflora in the silage-like fermentation mainly comprises lactobacilli and yeasts, creating a selective environment that promotes an increase in yeast proteins [6]. *L. amylovorus* has been shown to have a positive influence on yeast growth rate and ethanol yield, suggesting its potential to enhance the efficiency of sugarcane bioethanol fermentation [7]. The use of *L. amylovorus* DSM 19280 in the production of gluten-free sourdough bread has led to improvements in its nutritional value, quality, safety, and shelf life [8]. Several strains of *L. amylovorus* have been found to improve the quality of fermented feed [9], which has led to its widespread use as an inoculant in silage fermentation. Certain strains of this species have been isolated from other plants or plant-derived materials, such as tropical fruits [10], tomato pomace silage [11], fermented lemons [12], and corn steep liquor, which is a by-product of starch production from corn [13].

Intriguingly, *L. amylovorus* strains have also been found in diverse animal-related environments, including the intestinal tracts of swine and cattle, as well as in the oral cavity and faeces of humans [14,16]. *L. amylovorus* is particularly associated with pigs, including wild boars [17], and is detected with high frequency in the intestinal tract of piglets [18]. According to a study involving a probiotic formulation for pigs, *L. amylovorus* DSM 16698 was able to prevent infections caused by *Escherichia coli* and enhance body weight gain in pigs [19]. Certain *L. amylovorus* strains are known to have probiotic effects in humans. For example, a clinical study using a commercially available beverage containing *L. amylovorus* CP1563 reported that it prevents obesity by activating peroxisome proliferator-activated receptor α, which is involved in lipid metabolism [20,21].

Thus, *L. amylovorus* is adaptable to a wide range of ecological environments, including plants and animals, and is a promising lactic acid bacterium for commercial use (health, food, feed, and livestock production). As part of a project that aims to isolate *L. amylovorus* strains with economically useful traits and elucidate their biodiversity and host adaptation from an ecological perspective, we isolated seven *L. amylovorus* strains [two strains (BF125^T^ and BF186) from bovine faeces, one strain (TKL145) from porcine faeces, three strains (YK3, YK6, and YK10) from spent mushroom substrates (SMS), and one strain (NSK) from steeping water of a corn starch production plant (CPP)] and characterized them using a polyphasic approach to determine their taxonomic position.

## Isolation and ecology

Strains BF125^T^ and BF186 were isolated from fresh faeces of Japanese black beef cattle reared in an experimental field at the Institute of Livestock and Grassland Science, NARO (Nasushiobara, Tochigi, Japan; longitude 139° 55′ 49″, latitude 36° 55′ 9″). The strain TKL145 was isolated from a faecal sample of weanling piglets raised in an experimental facility at the Institute of Livestock and Grassland Science, NARO (Tsukuba, Ibaraki, Japan; longitude 140° 7′ 19″, latitude 36° 1′ 16″). Immediately after sampling, serial 10-fold dilutions of the faecal homogenates were prepared using PBS (pH 7.4). The diluted samples were streaked onto de Man, Rogosa, and Sharpe (MRS; Difco agar plates (pH 6.3) containing 1.5% (w/v) agar. After 3 days of incubation at 37 °C in an O_2_-free environment containing N_2_/H_2_/CO_2_ (80/10/10%) in an ANX-1 TE-HER Hard Anaerobox (Hirasawa), strains BF125^T^ and BF186 were isolated from faecal samples collected from different cattle on different days with four other *L. amylovorus*-like strains. These *L. amylovorus*-like strains were isolated from three of eight Japanese black beef cattle. Strain TKL145 was isolated after incubation for 3 days at 37 °C under anaerobic conditions (Anaero-Pack; Mitsubichi Gas Chemical).

Strains YK3, YK6 and YK10 were isolated from a corn cob-based SMS collected from a temporary storage location in Hara-mura, Nagano, Japan (longitude 138° 16′ 7″, latitude 35° 58′ 45″) together with other lactic acid bacterial strains [22,23]. The three strains were isolated from the homogenates of SMS following incubation on MRS agar plates for 72 h at 30 °C in an anaerobic chamber. Strain NSK was isolated from the steeping water of a CPP in Fuji, Shizuoka, Japan (longitude 138° 41′ 44″, latitude 35° 8′ 52″). The isolation conditions were as follows: after diluting the steeping water with PBS (pH 7.4), the isolates were inoculated on MRS agar plates and incubated at 37 °C for 48 h under anaerobic conditions (Anaero-Pack). The purified colonies were stored at −80 °C with 20% (v/v) dimethyl sulphoxide until analysis.

## 16S rRNA gene phylogeny

The 16S rRNA genes for these strains were amplified via PCR using primers 27F and 1492R [24] and sequenced as described previously [25]. The 16S rRNA sequences (nearly full-length) of all the isolates were compared with those of the type strains of the valid species using the EzBioCloud database [26]. The similarity values of the 16S rRNA gene sequences were calculated using pairwise nucleotide sequence alignment [26]. Based on similarities in the 16S rRNA gene sequences, the seven isolates were affiliated with the genus *Lactobacillus*; they showed the highest sequence similarities to *L. amylovorus* DSM 20531^T^ (accession no. AZCM01000082; 99.93–100%).

The obtained 16S rRNA sequences were aligned with publicly available sequences of the type strains of closely related species and reference strains of *L. amylovorus* using mafft (version 7.520) [27] in auto mode. The multiple sequence alignment was trimmed using trimAl (version 1.4.1) with the parameter ‘-gappyout’ [28]. A neighbour-joining (NJ) tree based on Kimura’s two-parameter model [29] was reconstructed using RapidNJ (version 2.3.2) [30]. The reliability of the tree topology was evaluated using bootstrap analysis with 1  000 replications. The 16S rRNA phylogenetic tree was visualized using Interactive Tree Of Life (iTOL) (version 6) [31].

The 16S rRNA phylogenetic analysis revealed that the seven isolates were in a clade containing reference strains and the type strain of *L. amylovorus* (Fig. S1, available in the online version of this article), suggesting that all isolates could be identified as *L. amylovorus*. Strains YK3, YK6, YK10, and NSK belonged to an intraspecific group represented by *L. amylovorus* DSM 20531^T^ (group A), which was separated from another group, named group B. Group B included strains BF125^T^, BF186, TKL145 and other references isolated from various animal origins, with strong bootstrap value of 99%. To clarify their taxonomic positions, a comparison based on whole genome sequences is presented in the following section.

## Genome features

Genomic DNA was extracted from strains BF125^T^, BF186, TKL145, YK3, YK6, YK10, and NSK for whole-genome sequence analysis using Illumina MiSeq or NovaSeq 6000 technology, as previously reported [32,33]. After filtering low-quality reads and trimming adapter sequences using fastp (version 0.23.4) [34], draft genomes were assembled using skesa (version 2.4.0) [35] and annotated using the dfast pipeline (version 1.2.0) [36]. Genomic quality was evaluated using the CheckM software (maker set, *Lactobacillus*) [37] in dfast. Summary statistics for the genome sequences are presented in Table S1.

The acquisition of relevant genomes from the National Center for Biotechnology Information Assembly Database was achieved through the utilization of genome_updater (version 0.6.3) (https://github.com/pirovc/genome_updater) [38]. Subsequently, the prediction and classification of clusters of orthologous groups (COGs) associated with protein-coding sequences were investigated using Pan-genome Explorer [39] in the 54 genomes of *L. amylovorus* (Fig. S2, Tables S1 and S2).

The protein-coding genes of *L. amylovorus* were clustered into 20 COG categories (Fig. S3). Although the COG ratio was largely similar between groups A and B, group A exhibited a higher ratio of defence mechanisms (V), energy production and conversion (C), coenzyme transport and metabolism (H), secondary metabolites biosynthesis, transport, and catabolism (Q), and inorganic ion transport and metabolism (P), and a lower ratio of nucleotide transport and metabolism (F), carbohydrate transport and metabolism (G), and replication, recombination, and repair (L) than group B. The most abundant COG category in the genomes of group A was amino acid transport and metabolism (E; 13.6±0.5%), followed by general function prediction only (R; 11.0±0.7%), carbohydrate transport and metabolism (G; 10.2±0.8%), and replication, recombination, and repair (L; 9.0±0.6%). The most common COG categories in group B genomes were amino acid transport and metabolism (E; 13.1±1.0%), carbohydrate transport and metabolism (G; 11.2±0.8%), general function prediction only (R; 10.7±0.8%), and replication, recombination and repair (L; 10.0±0.9%).

**Fig. 1.**
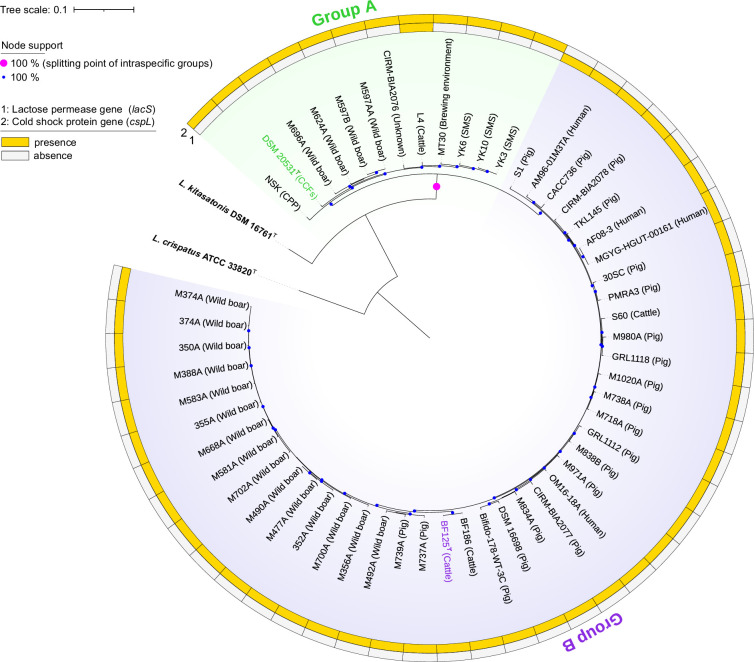
Phylogenetic tree reconstructed via concatenated alignment of 796 core genes based on whole-genome sequences of various strains of *Lactobacillus amylovorus* (*n*=54) including the isolates BF125^T^, BF186, TKL145, YK3, YK6, YK10, and NSK, and the type strains of the closely related species of the genus *Lactobacillus*. Strains DSM 20531^T^ (in the intraspecific group A of *L. amylovorus*) and BF125^T^ (in group B) are green- and purple-coloured, respectively. Isolation sources are shown in parentheses after each strain name. Outer rings display the presence (orange-coloured)/absence (light grey-coloured) status for genes of lactose permease (*lacS*) and cold shock protein (*cspL*). Local support of 100% calculated using the Shimodaira–Hasegawa test (based on 1000 replications) is indicated on the nodes with solid circle filled by light blue and magenta (splitting point of intraspecific groups between A and B). *Lactobacillus crispatus* ATCC 33820^T^ serves as an outgroup. The source of genomic sequence data is detailed in Tables S1 and S2. Bar, 0.1 substitutions per nucleotide position. CCFs, cattle waste-corn fermentations; CPP, corn starch production plant; SMS, spent mushroom substrates.

Phylogenetic analysis based on 796 conserved core genes was conducted using the PanACoTA pipeline (version 1.2.0) [40]. The maximum-likelihood core genome tree based on the GTR+CAT model and the discrete gamma model with 20 rate categories (Gamma20) was reconstructed through FastTree (version 2.1.11) [41] using the Shimodaira–Hasegawa test [42] for branch support (1 000 replications), and visualized using iTOL [31]. The core genome phylogenetic tree revealed that the seven isolates were assigned to *L. amylovorus*, and that groups A and B formed distinct lineages within the same species, supported by 100% bootstrap value (Fig. 1). Similar to the results of 16S rRNA phylogeny, the type strain DSM 20531^T^ (isolated from CCFs [5]) and three strains isolated from SMS (YK3, YK6, and YK10 in the present study), NSK (isolated from steeping water of CPP) were in the same cluster (group A), while group B included strains isolated from various animal origins, namely BF125^T^ (bovine faeces, in the present study), BF186 (bovine faeces, in the present study), TKL145 (porcine faeces, in the present study), Bifido-178-WT-3C (porcine faeces, shown in the BioSample accession SAMN14558271), S60 (bovine nasopharynx [43]), and DSM 16698 (intestine of weaning piglets [44]). Strain DSM 16698 was first proposed as a novel species in the genus ex-*Lactobacillus*, *Lactobacillus sobrius*, which is abundant in the intestine of weanling piglets and closely related to *L. amylovorus* [44]. The original classification of the strain DSM 16698 was established by a low value of the classical DNA–DNA hybridization (DDH; 49%), the ability to utilize raffinose and fructo-oligosaccharides, and the ability to grow at 45°C as compared to the type strain of *L. amylovorus*. However, a later study reclassified *L. sobrius* as a synonym of *L. amylovorus* based on the >70% threshold value of the classical DDH experiments (>79%) [45]. Our results and previous findings provide insights into the phenotypic and genotypic diversity of *L. amylovorus*.

**Fig. 2.**
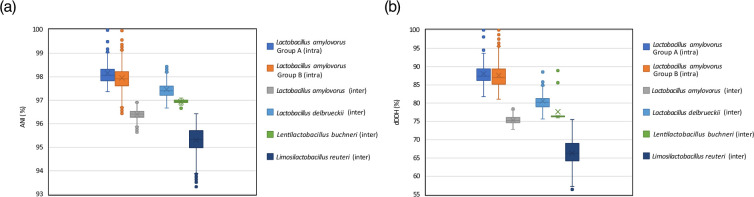
Pairwise ANI (**a**) and dDDH (**b**) percentages of intra- and inter-groups of *Lactobacillus amylovorus* genomes. These values were calculated using the 54 *L*. *amylovorus* genomes listed in Tables S1 and S2. For comparison, the ANI and dDDH values for inter-subspecies differences in the genus (ex-) *Lactobacillus* were determined using identical genomic datasets previously employed in taxonomic reports for *L. delbrueckii* (*n*=20) [71], *L. buchneri* (*n*=7) [51], and *L. reuteri* (*n*=33) [52].

To determine the exact taxonomic positions of the seven isolates, ANI based on blast was calculated using JSpeciesWS [46], and digital DNA–DNA hybridization (dDDH) was performed using Genome-to-Genome Distance Calculator 3.0 [47] with a dataset containing publicly available 54 genomes of *L. amylovorus* (Figs S4 and S5). The seven isolates were identified as a single *L. amylovorus* species because they had ANI values greater than the 95–96% species boundary [48] against the type strain DSM 20531^T^. The ANI values within groups A and B ranged from 97.37 to 99.99% and from 96.44 to 100.00%, respectively, whereas the intergroup ANI values ranged from 95.64–96.92%. Tanizawa *et al*. evaluated ANI values among six subspecies of *Lactobacillus delbrueckii*, which is a type species of the genus *Lactobacillus*, and found that they ranged from 97.2–98.4% [49]. Hence, the ANI values between groups A and B were lower than those observed among the *L. delbrueckii* subspecies, indicating that the two groups differ at the subspecies level. Because a cut-off value for ANI to separate subspecies of the genus (ex-) *Lactobacillus* has not been proposed, the ANI values between the two groups of *L. amylovorus* were compared with those of the inter-subspecies of the genus (ex-) *Lactobacillus*, such as *L. delbrueckii* (six subspecies [50]), *Lentilactobacillus buchneri* (two subspecies [51]), and *Limosilactobacillus reuteri* (six subspecies [52]). Results showed that the intergroup ANI values of *L. amylovorus* (96.39±0.19%; mean±SD) were lower than the inter-subspecies ANI values of *L. delbrueckii* (97.39±0.36%) and *L. buchneri* (96.93±0.11%), and higher than those of *L. reuteri* (95.30±0.59 %) (Fig. 2a), suggesting that the two groups should be distinguished based on subspecies criteria of the genus (ex-) *Lactobacillus*.

The dDDH analysis using the 54 genomes produced results consistent with the findings of the ANI analysis (Fig. 2B). Although an analysis of genome-sequenced strains from over 100 genera proposed a threshold of 79–80% dDDH for differentiating subspecies within bacteria and archaea [53], there is no valid threshold that is adaptable for subspecies of the genus *Lactobacillus*. The dDDH values between groups A and B ranged from 72.9 to 78.6%, falling within the values of other inter-subspecies of this genus (Fig. 2B), while being above the species cut-off value (70%) [54]. The dDDH value between strain BF125^T^ and type strain DSM 20531^T^ was 74.1%. Thus, it is strongly suggested that these two groups of *L. amylovorus* can be classified at the subspecies level.

We found a distinctive pattern in the presence/absence of two genes between the two groups (Fig. 1). The first gene was the cold shock protein gene (*cspL*) found in group A, but not in group B. Overexpression of *cspL* in *Lactiplantibacillus plantarum* NC8 temporarily ameliorated the decrease in growth rate caused by exposure to cold shock (8 °C), suggesting that *cspL* plays a role in adaptation to low temperatures [55]. Transgenic expression of *cspL* originating from the thermophilic bacterium *Bacillus coagulans* 2–6, has been shown to enhance heat tolerance and support the growth of various microorganisms, including *Escherichia coli* DH5α, *Saccharomyces cerevisiae* INVSc1, and *Pseudomonas putida* KT2440, by binding to a broad range of RNA species at high temperatures [56]. Thus, this cold shock protein is involved in stress response, mainly caused by the changing temperature of the external environment. In group A, three strains, YK3, YK6, and YK10, were isolated from SMS collected from a temporary outdoor storage [22,23], and strain NSK was isolated from steeping water of CPP, which is generally set at a high temperature (50 °C) [57]. Group B strains were isolated from the intestinal tracts of homeothermic mammals, such as humans, pigs, and cows, which have environments without large temperature fluctuations. Several strains in group A were isolated from eutherian wild boars and cattle, which have the possibility of natural contact with plant materials or fermented feeds, but in lower proportions than those in group B. A genomic study on intestinal commensals, particularly *Bifidobacterium* species, demonstrated a considerable decrease in heat shock response genes compared with their environmental counterparts [58]. The authors of this report suggested that this reduction is likely due to the organisms’ adaptation to the relatively stable temperatures present in specific ecological niches such as the human gut. Hence, maintaining the *cspL* might be essential to the capacity of group A to adapt to various environmental conditions, particularly those with potential temperature fluctuations.

The second characteristic gene was the lactose permease gene (*lacS*), which encodes the membrane protein responsible for lactose transport [59]. Lactose is the major carbohydrate in eutherian milk [60], and many lactic acid bacteria take up lactose as a free sugar via lactose permease and use it as an energy source [61]. Group B (42 sets), whose members were all eutherian isolates, retained *lacS*, whereas group A (12 sets) retained it as a fragmented *lacS* (pseudogenes), except for the L4 strain. Thus, *lacS* may have become an essential gene for members of group B owing to their adaptation to the eutherian intestinal environment, as evidenced by the high percentage of *L. amylovorus* found in piglets [18]. *L. delbrueckii* subsp. *bulgaricus*, which is used for industrial yoghurt production, demonstrated various gene fragmentations and genome shrinkage compared to other subspecies of *L. delbrueckii*, likely as a result of adaptation to the milk environment as well as increased specificity in carbohydrate fermentation [62]. The fragmentation of *lacS* observed in group A may be a part of the adaptation process from the eutherian intestinal environment to another, and that strain L4 may be in the process of transition. These results also suggested that the mechanisms underlying lactose uptake and tolerance to temperature stress may be important factors in the diversity and host adaptation of *L. amylovorus*. Further studies on *L. amylovorus* isolated from a wide range of environments would be useful for elucidating the evolutionary processes and mechanisms of adaptation to various environments.

## Physiology and chemotaxonomy

Bacterial morphology was observed after 2 days of incubation at 37 °C under anaerobic conditions (AnaeroPack-Anaero, Mitsubishi Gas Chemical Company) using a light microscope (BA210E, Shimadzu RIKA Corporation). Gram staining was performed using a Gram Stain Kit (ScyTek Laboratories, Inc.). Spore formation was confirmed using the Scheffer–Fulton-modified Wirtz staining method (Wirtz Stain Kit, Muto Pure Chemicals, Ltd.). The colony morphology and size were observed after incubation on MRS agar plates for 2 days at 37 °C under anaerobic conditions. The appearance of the culture was observed after incubation in MRS broth for 3 days at 37 °C. The presence of gas produced from glucose in MRS medium was determined using Durham tubes. Dextran production was observed in MRS agar medium with sucrose (5%) replacing glucose as the carbon source, and colony status was observed. Catalase activity was assessed by spreading colonies grown on MRS agar medium on glass slides, adding 3% (v/v) H_2_O_2_, and observing the presence of bubbles. The oxidase activity was determined using a cytochrome oxidase test strip ‘Nissui’ (Nissui Pharmaceutical Co. Ltd.), according to the manufacturer’s instructions. The lactic acid configuration was analysed via an enzymatic method using an F-kit d-lactic acid/l-lactic acid kit (JK International, Inc.). After the bacteria were incubated statically for 48 h in MRS broth at various pH (pH 3.0, 4.0, 5.0, 6.0, 7.0, 8.0, 9.0, and 10.0), NaCl concentrations [0, 1, 2, 3, 4, 5, 6, 7, 8, 9, and 10% (w/v)], or temperature conditions (15, 20, 25, 30, 37, 45, and 50 °C), the optical density at 660 nm and the culture pH were measured to determine pH tolerance, salt tolerance, and temperature growth. Physiological and biochemical characteristics were analysed using API 50 CH and API ZYM strips (bioMérieux) in duplicate at 37 °C according to the manufacturer’s instructions. The fatty acid profiles derived from the bacteria cultivated on MRS agar plates at 37 °C for 2 days were analysed using the Sherlock Microbial Identification System (midi; version 6.0; database: moore6) by the identification service of Techno Suruga Laboratories.

Although the fatty acid compositions of strains BF125^T^, DSM 16698, and YK10 were relatively similar, differences were evident in strains NSK and DSM 20531^T^, with C_14 : 0_ being the major component. However, no particular fatty acid served as a taxonomically useful marker for intraspecific differentiation (Table S3). The major fatty acids detected in strain BF125^T^ were C_18 : 1_ ω9*c* (31.3%), C_16 : 0_ (22.7%), C_19_ cyclopropane 9, 10 (16.7%), and summed feature 10 (15.0%). All the tested broth cultures in group B appeared clear because of the complete settling of the bacteria, whereas all cultures in group A appeared slightly turbid with incomplete bacterial sedimentation. In addition to the genotypic evidence, the phenotypic differences between the two groups are summarized in Table 1.

**Table 1.** Differential phenotypic features and isolation sources of tested strains belonging to *Lactobacillus amylovorus* subsp. *amylovorus* or *Lactobacillus amylovorus* subsp. *animalis* Strains in group A (*L. amylovorus* subsp. *amylovorus*): 1, DSM 20531^T^; 2, YK3; 3, YK6; 4, YK10; 5, NSK. Strains in group B (*L. amylovorus* subsp. *animalis*): 6, BF125^T^; 7, BF186; 8, DSM 16698; and 9, DSM 107288 (=Bifido-178-WT-3C); 10, TKL145. All tested characteristics were investigated under identical conditions in the present study. Orange-coloured, positive; light grey-coloured, negative.

All the tested strains in group A were able to ferment salicin and gentiobiose, whereas those in group B could not. Salicin is a β-glucoside found in several species of *Salix* and *Populus* [63], and β-glucoside is originally known in nature as a plant metabolite and is abundant in plant bodies [64]. Gentiobiose is a β-linked glucobiose found in gentian root [65], indicating that both salicin and gentiobiose are highly related to plants. Considering that the tested strains of group A were isolated from plant-related sources, the ability to utilize these plant-related carbohydrates could play a significant role in the environmental adaptation of group A. β-Glucosides are molecules composed of a glucose unit connected either to a non-sugar component, such as salicin, or to another glucose unit, such as gentiobiose [66]. Some Lactobacilli can degrade plant-derived β-glucosides, facilitated by the activity of phospho-β-glucosidases [67,68]. Metabolic characterization of *L. acidophilus* NCFM demonstrated that certain phospho-β-glucosidases are critical in plant β-glucosides hydrolysis, including salicin and gentiobiose [68]. Among the genes encoding phospho-β-glucosidases found in *L. amylovorus* genomes (*n*=54), a certain phospho-β-glucosidase gene found in the group B genomes formed a cluster separate from the other strains (Fig. S6 and Table S4). Although the taxonomic separation of this enzyme was observed at the amino acid level (Fig. S7), further investigation is necessary to clarify the dissimilarities in its characteristics between groups A and B.

All tested members of group B differed uniquely from those of group A in terms of being positive for acid production from lactose, consistent with the findings of the genomic analysis of the presence/absence of the *lacS* gene (Fig. 1). Members of group B were isolated from various sources related to animals in the Eutheria group, as described above, suggesting that this group is more likely to be related to animals. Thus, groups A and B can be differentially characterized by their fermentability. Several variable reactions in each group were observed as follows; (i) d-mannose, d-mannitol, methyl α-d-glucopyranoside, melibiose, raffinose, glycogen, and turanose assimilation in group A; (ii) d-galactose, d-mannose, and raffinose assimilation in group B; (iii) production of cystine arylamidase, α-galactosidase, and β-galactosidase in group A; (iv) production of esterase lipase (C8), α-galactosidase in group B. These findings suggest that these features are strain-specific and that all isolates obtained in this study are not clonal. Detailed physiological and biochemical characteristics are provided in the taxonomic descriptions below.

Based on the results of this polyphasic approach, we propose that *L. amylovorus* can be divided into two subspecies. We propose the creation of *Lactobacillus amylovorus* subsp. *animalis* subsp. nov. with the type strain BF125^T^ (=MAFF 212522^T^=DSM 115528^T^), and the automatic creation of *Lactobacillus amylovorus* subsp. *amylovorus* subsp. nov. with the type strain ATCC 33620^T^ (=CCUG 27201^T^=CIP 102989^T^=DSM 20531^T^=JCM 1126^T^=LMG 9496^T^=NCAIM B.01458^T^=NRRL B-4540^T^). Based on the results of the newly reported characteristics in the present study, an emended description of *Lactobacillus amylovorus* Nakamura 1981 [45] has also been provided.

## Emended description of *Lactobacillus amylovorus* Nakamura 1981

*Lactobacillus amylovorus* (a.my.lo.vo’rus. Gr. neut. n. *amylon*, starch; L. v. *vorare*, to devour; N.L. masc. adj. *amylovorus*, starch-devouring).

The following characteristics emended those reported in the original description by Nakamura [5] and in a list of species properties by Zheng *et al*. [3].

The bacterium is Gram-stain-positive, non-motile, non-spore-forming, catalase-negative, oxidase-negative, facultatively anaerobic, homofermentative, and rod-shaped. The bacteria grow individually and in short chains. The agar colonies are white, convex, smooth, circular, complete, and opaque. The broth showed variable appearance after incubation at 37 °C for 3 days: some samples were turbid, others clear owing to the settling of the bacteria. They produce both d- and l-lactate and small amounts of acetate but no gas from glucose or gluconate. Growth temperatures are as follows: optimum, 37–45 °C; minimum, 20–25 °C; and maximum, 45–50 °C. In the API 50 CH test system (incubation at 37 °C for 2 days), acids are produced from d-glucose, d-fructose, *N*-acetyl-glucosamine, aesculin ferric citrate, and maltose, but not from glycerol, erythritol, d-arabinose, l-arabinose, d-ribose, d-xylose, l-xylose, d-adonitol, methyl β-d-xylopyranoside, l-sorbose, l-rhamnose, dulcitol, inositol, d-sorbitol, methyl α-d-mannopyranoside, inulin, melezitose, xylitol, d-lyxose, d-tagatose, d-fucose, l-fucose, d-arabitol, l-arabitol, gluconate, 2-keto-gluconate, and 5-keto-gluconate. Variable reactions for acid production from d-galactose, d-mannose, d-mannitol, methyl α-d-glucopyranoside, amygdalin, arbutin, salicin, cellobiose, lactose, melibiose, sucrose, trehalose, d-raffionose, starch, glycogen, gentiobiose, and turanose are observed. In the API ZYM test system, positive reactions are observed for esterase (C4), leucine arylamidase, valine arylamidase, acid phosphatase, naphthol-AS-BI-phosphohydrolase, α-glucosidase, and β-glucosidase. Negative reactions are obtained from alkaline phosphatase, lipase (C14), trypsin, α-chymotrypsin, β-glucuronidase, *N*-acetyl-β-glucosaminidase, α-mannosidase, and α-fucosidase. Various reactions are obtained from esterase lipase (C8), cystine arylamidase, and α-galactosidase. An extracellular amylolytic enzyme is then formed. Nitrate is not reduced to nitrite. Nicotinic acid, pantothenic acid, folic acid, and riboflavin are essential for growth, whereas thiamine is not required. C_14 : 0_, C_16 : 0_, C_18 : 1_ ω9*c*, and C_19_ cyclopropane 9, 10 are the major fatty acids found in strain DSM 20531^T^. The genome size of the type strain is 2.02 Mbp, and the G+C content of the DNA is 37.8 mol% (whole-genome analysis). This micro-organism is a characteristic representative of the swine intestinal microbiota [69,70] and has been isolated from other environments, such as sourdough, cattle waste-corn fermentation, spent mushroom substrates, steeping water of corn starch production plant, and bovine faeces. The type strain is ATCC 33620^T^ (=CCUG 27201^T^=CIP 102989^T^=DSM 20531^T^=JCM 1126^T^=LMG 9496^T^=NCAIM B.01458^T^=NRRL B-4540^T^), isolated from cattle waste-corn fermentation. The GenBank/EMBL/DDBJ accession number for the 16S rRNA gene sequence of strain DSM 20531^T^ is AY944408. The genome sequence accession number for strain DSM 20531^T^ is AZCM00000000.

## Description of *Lactobacillus amylovorus* subsp. *amylovorus* subsp. nov.

*Lactobacillus amylovorus* subsp. *amylovorus* (a.my.lo.vo’rus. Gr. neut. n. *amylon*, starch; L. v. *vorare*, to devour; N.L. masc. adj. *amylovorus*, starch-devouring).

The description is essentially in agreement with that given above for the species *Lactobacillus amylovorus*, with the following modifications.

In the API 50 CH test system (incubation at 37 °C for 2 days), acids are produced from d-galactose, d-glucose, d-fructose, *N*-acetyl-glucosamine, aesculin ferric citrate, salicin, maltose, trehalose, and gentiobiose, but not from glycerol, erythritol, d-arabinose, l-arabinose, d-ribose, d-xylose, l-xylose, d-adonitol, methyl β-d-xylopyranoside, l-sorbose, l-rhamnose, dulcitol, inositol, d-sorbitol, methyl α-d-mannopyranoside, lactose, inulin, melezitose, xylitol, d-lyxose, d-tagatose, d-fucose, l-fucose, d-arabitol, l-arabitol, gluconate, 2-keto-gluconate, and 5-keto-gluconate. Variable reactions are observed for acid production from d-mannose, d-mannitol, methyl α-d-glucopyranoside, amygdalin, arbutin, cellobiose, melibiose, sucrose, raffinose, starch, glycogen, and turanose. This proposed subspecies ferments several β-glucosides including aesculin, salicin and gentiobiose with variable reactions for arbutin, amygdalin and cellobiose. In the API ZYM test system, positive reactions are observed for esterase (C4), leucine arylamidase, valine arylamidase, acid phosphatase, naphthol-AS-BI-phosphohydrolase, α-glucosidase, and β-glucosidase. Negative reactions are obtained from alkaline phosphatase, esterase lipase (C8), trypsin, α-chymotrypsin, *N*-acetyl-β-glucosaminidase, α-mannosidase, and α-fucosidase. The reactions of the following enzymes vary: lipase (C14),cystine arylamidase, α-galactosidase, β-galactosidase, and β-glucuronidase. The type strain is ATCC 33620^T^ (=CCUG 27201^T^=CIP 102989^T^=DSM 20531^T^=JCM 1126^T^=LMG 9496^T^=NCAIM B.01458^T^=NRRL B-4540^T^), isolated from cattle waste-corn fermentation. At least four additional strains (YK3, YK6, YK10, and NSK) are included in this subspecies.

## Description of *Lactobacillus amylovorus* subsp. *animalis* subsp. nov.

*Lactobacillus amylovorus* subsp. *animalis* (a.ni.ma’lis. L. gen. n. *animalis*, of a living being, an animal).

The bacterium is Gram-stain-positive, non-motile, non-spore-forming, catalase-negative, cytochrome oxidase-negative, facultatively anaerobic, homofermentative, and rod-shaped. The bacteria are 0.5–0.7 µm wide and 3.0–7.0 µm long. Colonies grown on MRS agar plates at 37 °C for 3 d under anaerobic conditions are 1.0–2.0 mm in diameter and milky white, round, and glossy in appearance. This strain does not produce any gas from glucose; both d- and l-lactate are produced as end products of glucose (D/L, 40 : 60). Growth occurs at 30–45 °C (optimum, 37 °C), pH 5.0–8.0 (optimum, pH 6.0), and with NaCl concentrations of 1.0–3.0% (w/v). In the API 50 CH test system (incubation at 37 °C for 2 days), acids are produced from d-glucose, d-fructose, *N*-acetyl-glucosamine, aesculin ferric citrate, maltose, lactose, sucrose, and starch, but not from glycerol, erythritol, d-arabinose, l-arabinose, d-ribose, d-xylose, l-xylose, d-adonitol, methyl β-d-xylopyranoside, l-sorbose, l-rhamnose, dulcitol, inositol, d-mannitol, d-sorbitol, methyl α-d-mannopyranoside, methyl α-d-glucopyranoside, amygdalin, arbutin, salicin, melibiose, inulin, melezitose, glycogen, xylitol, gentiobiose, turanose, d-lyxose, d-tagatose, d-fucose, l-fucose, d-arabitol, l-arabitol, gluconate, 2-keto-gluconate, and 5-keto-gluconate. Variable reactions have been observed for acid production from d-galactose, d-mannose, cellobiose,trehalose, and raffinose. For this proposed subspecies, aesculin citrate is the only β-glucoside that supports acid formation, but citrate is also a substrate for metabolism of many lactobacilli. In the API ZYM test system, positive reactions are obtained from esterase (C4), leucine arylamidase, valine arylamidase, cystine arylamidase, acid phosphatase, naphthol-AS-BI-phosphohydrolase, β-galactosidase, α-glucosidase, and β-glucosidase. Negative reactions are obtained from alkaline phosphatase, lipase (C14), trypsin, α-chymotrypsin, β-glucuronidase, *N*-acetyl-β-glucosaminidase, α-mannosidase, and α-fucosidase. The reactions of the following enzymes vary: esterase lipase (C8) and α-galactosidase. Dextran is not produced from sucrose, and ammonia is not produced from arginine. C_16 : 0_, C_18 : 1_ ω9*c*, C_19_ cyclopropane 9,10*,* and summed feature 10 are the major fatty acids in strain BF125^T^. The genome size of the type strain is 1.98 Mbp, and the G+C content of the DNA is 37.8 mol% (whole-genome analysis). The type strain, BF125^T^ (=MAFF 212522^T^=DSM 115528^T^), was isolated from bovine faeces. At least five additional strains [BF186, TKL145, Bifido-178-WT-3C (=DSM 107288), S60, AF08-3, and DSM 16698] are included in this subspecies. The GenBank/EMBL/DDBJ accession number for the 16S rRNA gene sequence of strain BF125^T^ is LC771959. The INSDC accession numbers for the draft genome sequences of strain BF125^T^ are BTFR01000001–BTFR01000033.

## Supplementary material

10.1099/ijsem.0.006517 Uncited Supplementary Material 1.

## Abbreviations

ANI: average nucleotide identity
CCF: cattle waste-corn fermentation
COG: cluster of orthologous group
CPP: corn starch production plant
dDDH: digital DNA–DNA hybridization
iTOL: interactive tree of life
MRS: de Man, Rogosa, and Sharpe
NJ: neighbour-joining
SMS: spent mushroom substrates
