# Corrigendum. Proposal of Lactobacillus amylovorus subsp. animalis subsp. nov. and an emended description of Lactobacillus amylovorus

**DOI:** 10.1099/ijsem.0.006564

**Published:** 2024-11-04

**Authors:** Kenji Yamane, Yasuhiro Tanizawa, Hisami Kobayashi, Tomomi Kamizono, Yoichiro Kojima, Hiroki Takagi, Masanori Tohno

**Affiliations:** 1 Innovative Animal Production System, University of Tsukuba, 1-1-1 Tennodai, Tsukuba, Ibaraki 305-8571, Japan; 2 Nihon Shokuhin Kako Co. Ltd, 30 Tajima, Fuji, Shizuoka 417-8530, Japan; 3 Department of Informatics, National Institute of Genetics, Mishima, Shizuoka 411-8540, Japan; 4 Institute of Livestock and Grassland Science, National Agriculture and Food Research Organization, Nasushiobara, Tochigi 329-2793, Japan; 5 Research Center of Genetic Resources, National Agriculture and Food Research Organization, Tsukuba, Ibaraki 305-8602, Japan

In the published version of the article, we proposed the name *Lactobacillus amylovorus* subsp. *animalis*. This name is illegitimate, as it contravenes Rule 12(b) of the International Code of Nomenclature of Prokaryotes [1]. As a replacement name, we propose *Lactobacillus amylovorus* subsp. *animalium*. The protologue is then as follows:

*Lactobacillus amylovorus* subsp. *animalium* (a.ni.ma′li.um. L. gen. pl. n. *animalium*, of animals).

The bacterium is Gram-stain-positive, nonmotile, non-spore-forming, catalase-negative, cytochrome oxidase-negative, facultatively anaerobic, homofermentative and rod-shaped. The bacteria are 0.5–0.7 µm in width and 3.0–7.0 µm in length. Colonies grown on de Man, Rogosa, and Sharpe (MRS; Difco, Detroit, MI, USA) agar plates at 37 °C for 3 days under anaerobic conditions are 1.0–2.0 mm in diameter and milky white, round and glossy in appearance. This strain does not produce any gas from glucose; both d- and l-lactate are produced as end products of glucose (d/l, 40 : 60). Growth occurs at a temperature of 30–45 °C (optimum 37 °C), pH of 5.0–8.0 (optimum pH 6.0) and NaCl concentration of 1.0–3.0% (w/v). In the API 50 CH test system (incubation at 37 °C for 2 days), acids are produced from d-glucose, d-fructose, *N*-acetyl-glucosamine, aesculin ferric citrate, d-maltose, d-lactose, d-sucrose and starch, but not from glycerol, erythritol, d-arabinose, l-arabinose, d-ribose, d-xylose, l-xylose, d-adonitol, methyl-*β*-d-xylopyranoside, l-sorbose, l-rhamnose, dulcitol, inositol, d-mannitol, d-sorbitol, methyl-*α*-d-mannopyranoside, methyl-*α*-d-glucopyranoside, amygdalin, arbutin, salicin, d-melibiose, inulin, d-melezitose, glycogen, xylitol, gentiobiose, d-turanose, d-lyxose, d-tagatose, d-fucose, l-fucose, d-arabitol, l-arabitol, gluconate, 2-keto-gluconate and 5-keto-gluconate. Variable reactions have been observed for acid production from d-galactose, d-mannose, d-cellobiose, d-trehalose and d-raffinose. For this proposed subspecies, aesculin citrate is the only *β*-glucoside that supports acid formation, but citrate is also a substrate for the metabolism of many lactobacilli. In the API ZYM test system, positive reactions are obtained from esterase (C4), leucine arylamidase, valine arylamidase, cystine arylamidase, acid phosphatase, naphthol-AS-BI-phosphohydrolase, *β*-galactosidase, *α*-glucosidase and *β*-glucosidase. Negative reactions are obtained from alkaline phosphatase, lipase (C14), trypsin, *α*-chymotrypsin, *β*-glucuronidase, *N*-acetyl-*β*-glucosaminidase, *α*-mannosidase and *α*-fucosidase. The reactions of the following enzymes vary: esterase lipase (C8) and *α*-galactosidase. Dextran is not produced from sucrose, and ammonia is not produced from arginine. C_16 : 0_, C_18 : 1_ ω*9c*, C_19_ cyclopropane 9,10 and summed feature 10 are the major fatty acids in strain BF125^T^. The genome size of the type strain is 1.98 Mbp, and the mol% G+C content of the DNA is 37.8 (whole-genome analysis). The type strain BF125^T^ (= MAFF 212522^T^ = DSM 115528^T^) is isolated from bovine faeces. At least five additional strains [BF186, TKL145, Bifido-178-WT-3C (= DSM 107288), S60, AF08-3, and DSM 16698] are included in this subspecies. The GenBank/The European Molecular Biology Laboratory (EMBL) /DNA Data Bank of Japan (DDBJ) accession number for the 16S rRNA gene sequence of strain BF125^T^ is LC771959. The International Nucleotide Sequence Database Collaboration (INSDC) accession numbers for the draft genome sequence of strain BF125^T^ are BTFR01000001−BTFR01000033.

We would like to thank Mr Meng-Syun Li (National Chung Hsing University) and Dr Aharon Oren (The Hebrew University of Jerusalem) for their contribution to this corrigendum. The authors apologize for any inconvenience caused.

## References

[1] Oren A, Arahal DR, Göker M, Moore ERB, Rossello-Mora R (2023). International Code of Nomenclature of Prokaryotes. Prokaryotic Code (2022 Revision). Int J Syst Evol Microbiol.

