# Supplementary material for: Proposal of Lactobacillus amylovorus subsp. animalis subsp. nov. and an emended description of Lactobacillus amylovorus
Source: Int J Syst Evol Microbiol. 2024 Sep 12;74(9):006517. doi: 10.1099/ijsem.0.006517 (PMC12453554; doi:10.1099/ijsem.0.006517)
Supplement: Uncited Supplementary Material 1. [file ijsem-74-06517-s001.pdf]

IJSEM supplementary materials for:

Proposal of *Lactobacillus amylovorus* subsp. *animalis* subsp. nov. and an emended description of *Lactobacillus amylovorus*

Author names
Kenji Yamane[1,2], Yasuhiro Tanizawa[3], Hisami Kobayashi[4], Tomomi Kamizono[4], Yoichiro Kojima[4], Hiroki Takagi[2] and Masanori Tohno[1,4,5]

Affiliation
[1]Innovative Animal Production System, University of Tsukuba, 1-1-1 Tennodai, Tsukuba, Ibaraki 305-8571, Japan; [2]Nihon Shokuhin Kako Co., LTD, 30, Tajima, Fuji, Shizuoka 417-8530, Japan; [3]Department of Informatics, National Institute of Genetics, Mishima, Shizuoka 411-8540, Japan; [4]Institute of Livestock and Grassland Science, National Agriculture and Food Research Organization, Nasushiobara, Tochigi 329-2793, Japan; [5]Research Center of Genetic Resources, National Agriculture and Food Research Organization, Tsukuba, Ibaraki 305-8602, Japan.

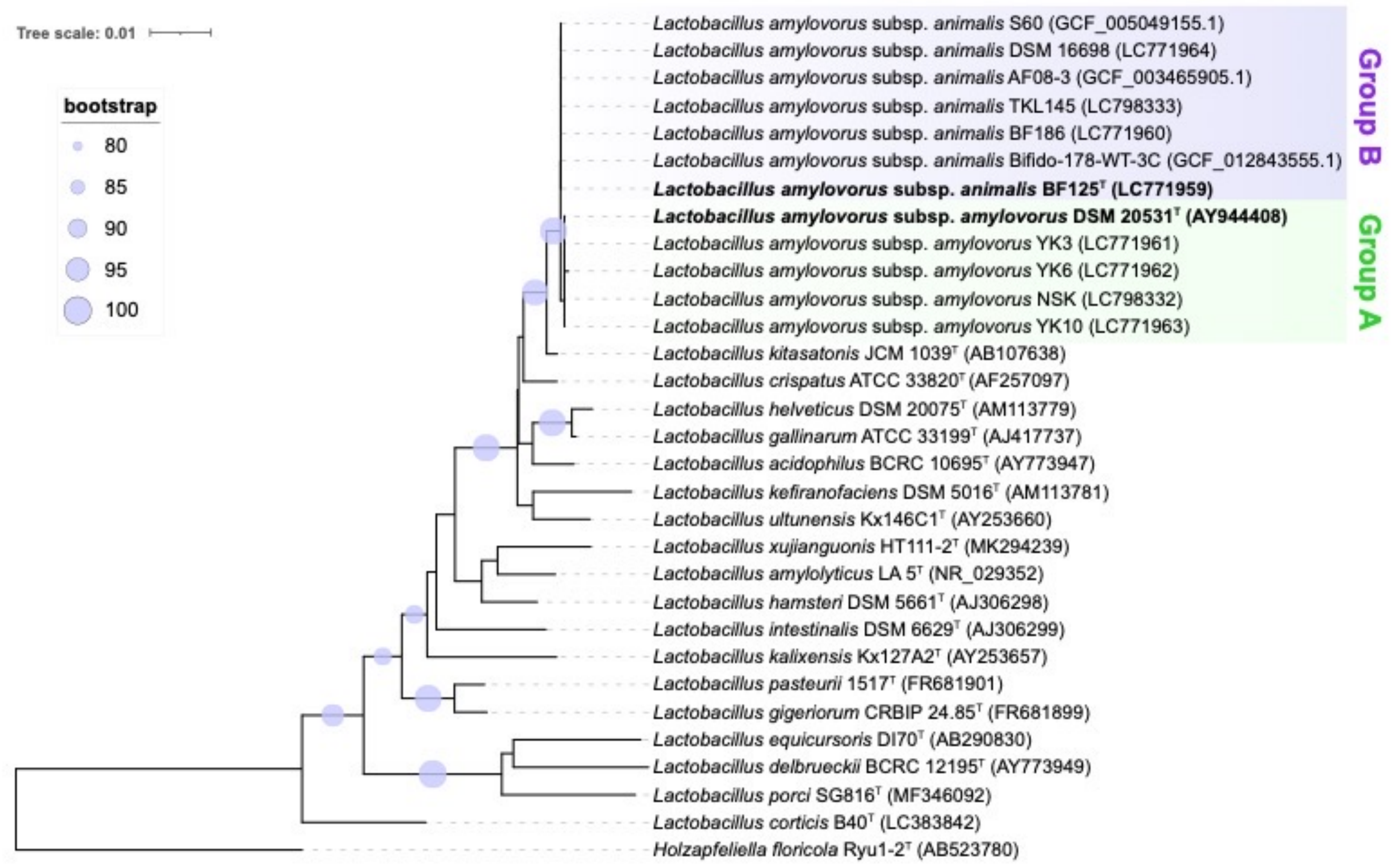

Supplementary Fig. S1. Neighbor-joining tree based on 16S rRNA gene sequences showing the phylogenetic relationship of seven isolates BF125^T, BF186, TKL145, YK3, YK6, YK10 and NSK within the closely related species of the genus *Lactobacillus*. Strains DSM 20531^T (representative of group A) and BF125^T (representative of group B) are highlighted in boldface type. The sequence of *Holzapfeliella floricola* Ryu1-2^T is used as an outgroup. Bootstrap percentages greater than 80% (based on 1,000 replications) are shown at branch points. Bar, 0.01 substitutions per nucleotide position.

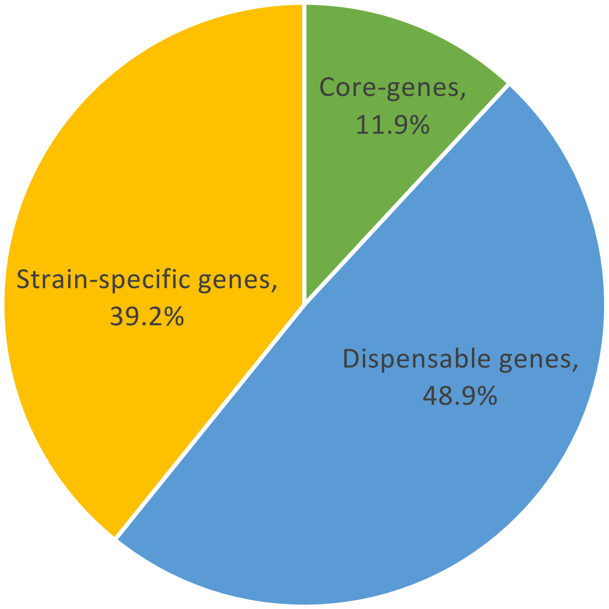

Supplementary Fig. S2. The pan-genome pie chart showing gene percentages of *L. amylovorus* in 54 strains visualized with the use of Pan-genome Explorer. Core genes accounted for 11.9%, dispensable genes for 48.9%, and strain specific genes for 39.2%.

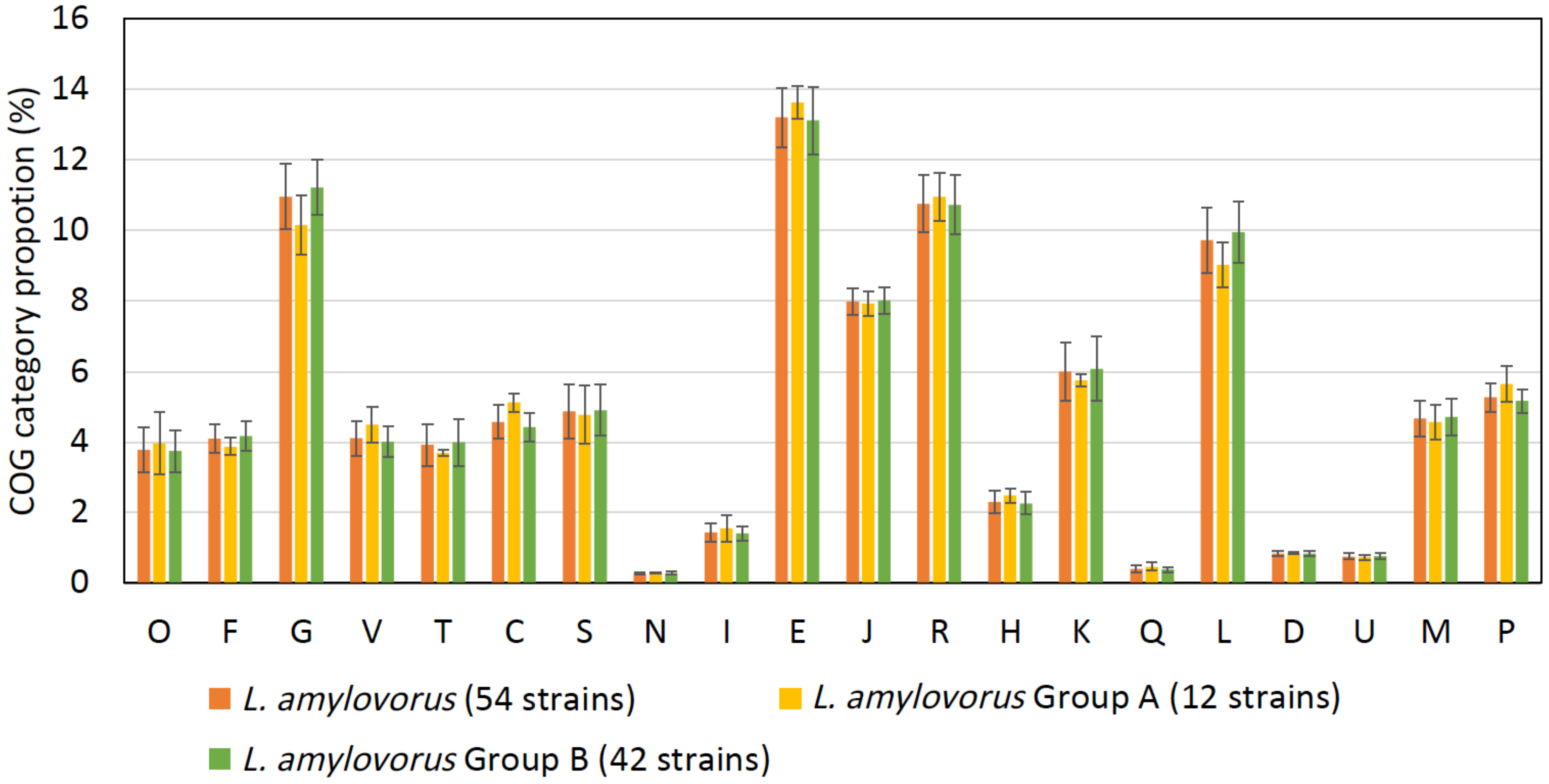

Supplementary Fig. S3. Proportions of genes associated with each clusters of orthologous groups (COG) category of *L. amylovorus* in 54 strains, group A (12 strains), and group B (42 strains). J, Translation, ribosomal structure and biogenesis; G, Carbohydrate transport and metabolism; U, Intracellular trafficking, secretion, and vesicular transport; D, Cell cycle control, cell division, chromosome partitioning; L, Replication, recombination and repair; I, Lipid transport and metabolism; Z, Cytoskeleton; M, Cell wall/membrane/envelope biogenesis; C, Energy production and conversion; Q, Secondary metabolites biosynthesis, transport and catabolism; B, Chromatin structure and dynamics; Y, Nuclear structure; W, Extracellar structures; N, Cell motility; R, General function prediction only; E, Amino acid transport and metabolism; K, Transcription; T, Signal transduction mechanisms; A, RNA processing and modification; O, Posttranslational modification, protein turnover, chaperones; S, Function unknown; F, Nucleotide transport and metabolism; H, Coenzyme transport and metabolism; P, Inorganic ion transport and metabolism; V, Defense mechanisms.

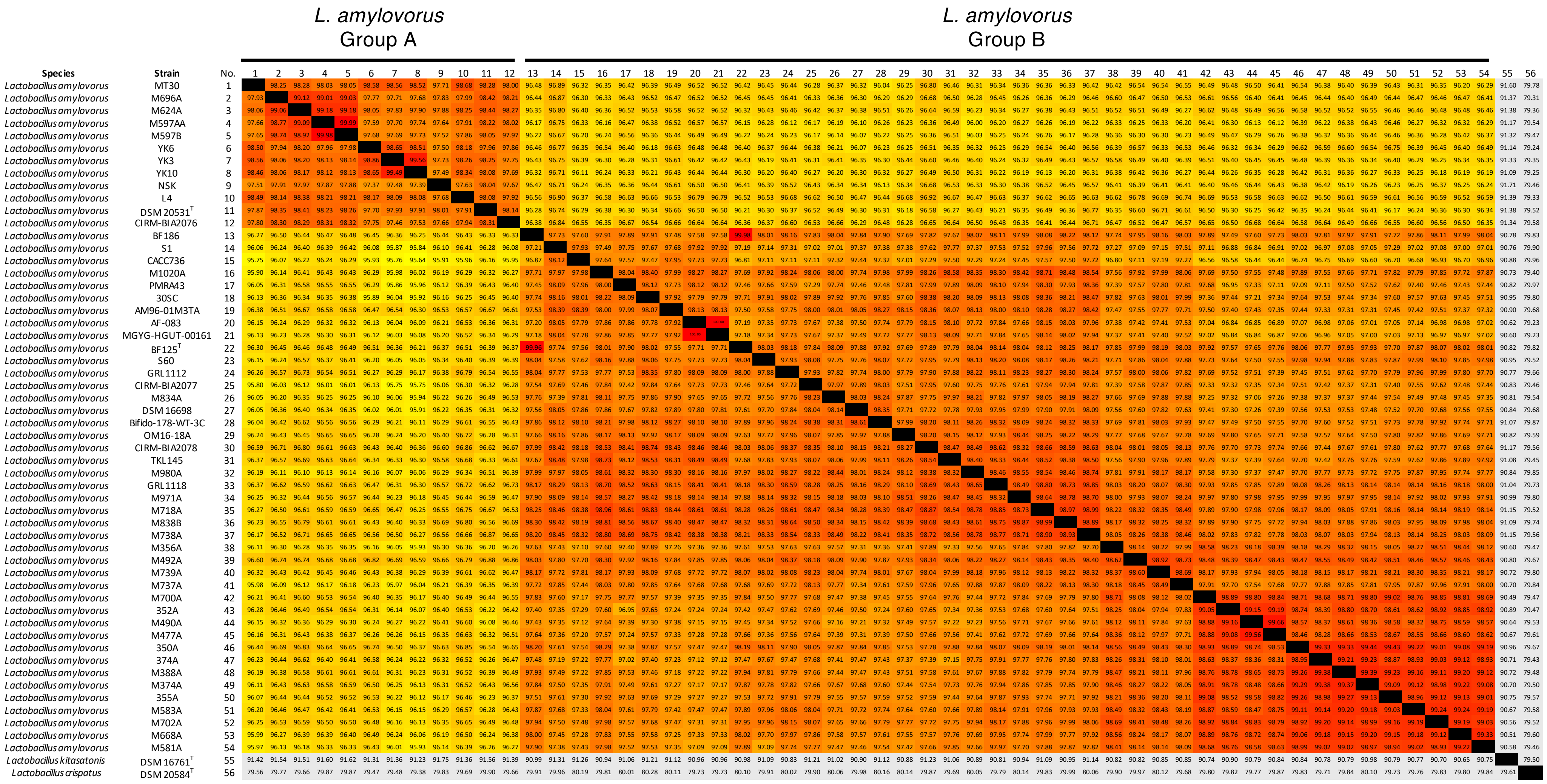

Supplementary Fig. S4. Pairwise comparison of ANI percentages among *L. amylovorus* and its closely related taxa. Group A consists of nos. 1 to 12, while group B comprises nos. 13 to 54.

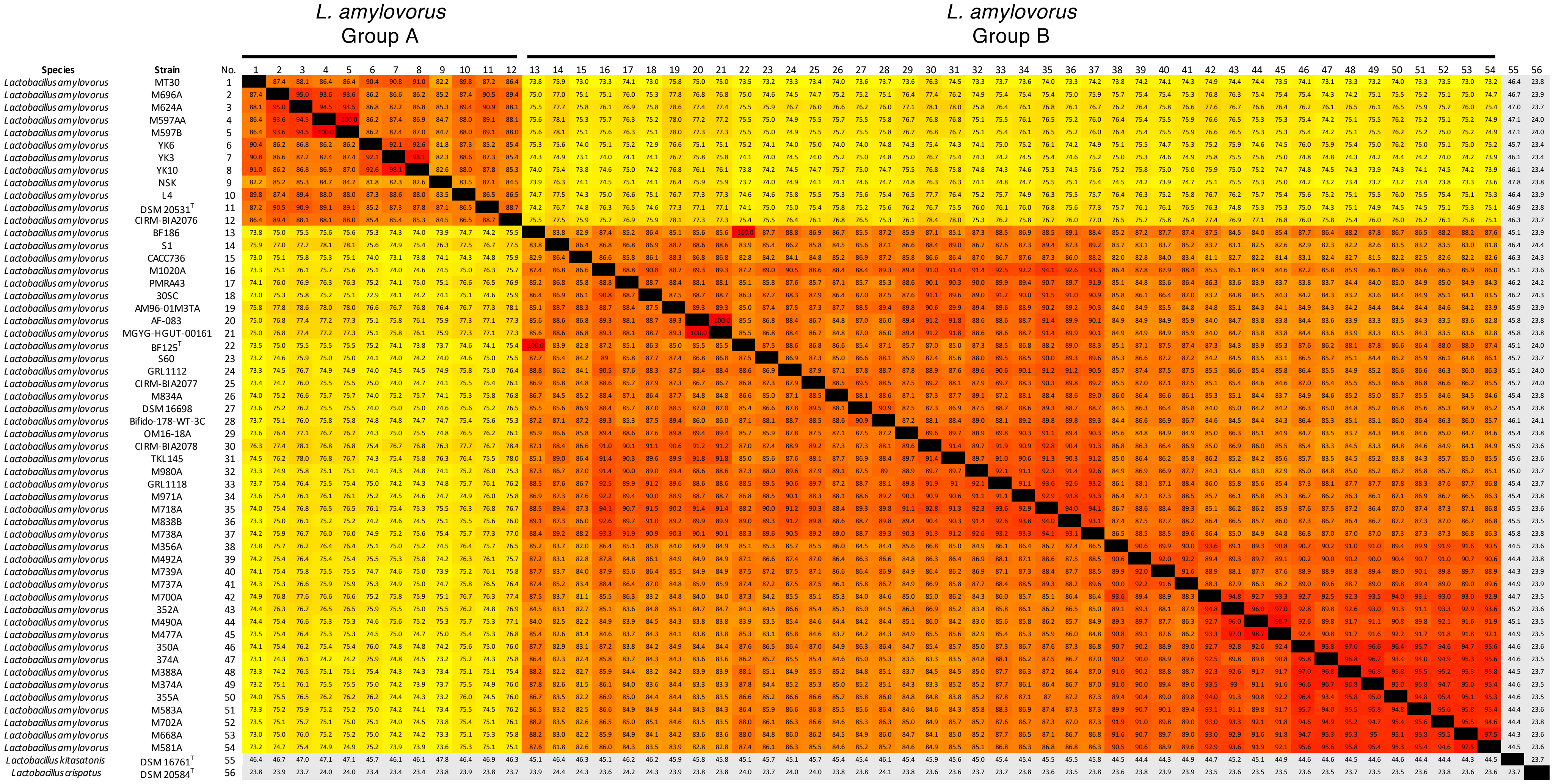

Supplementary Fig. S5. Pairwise comparison of dDDH percentages among *L. amylovorus* and its closely related taxa. Group A consists of nos. 1 to 12, while group B comprises nos. 13 to 54.

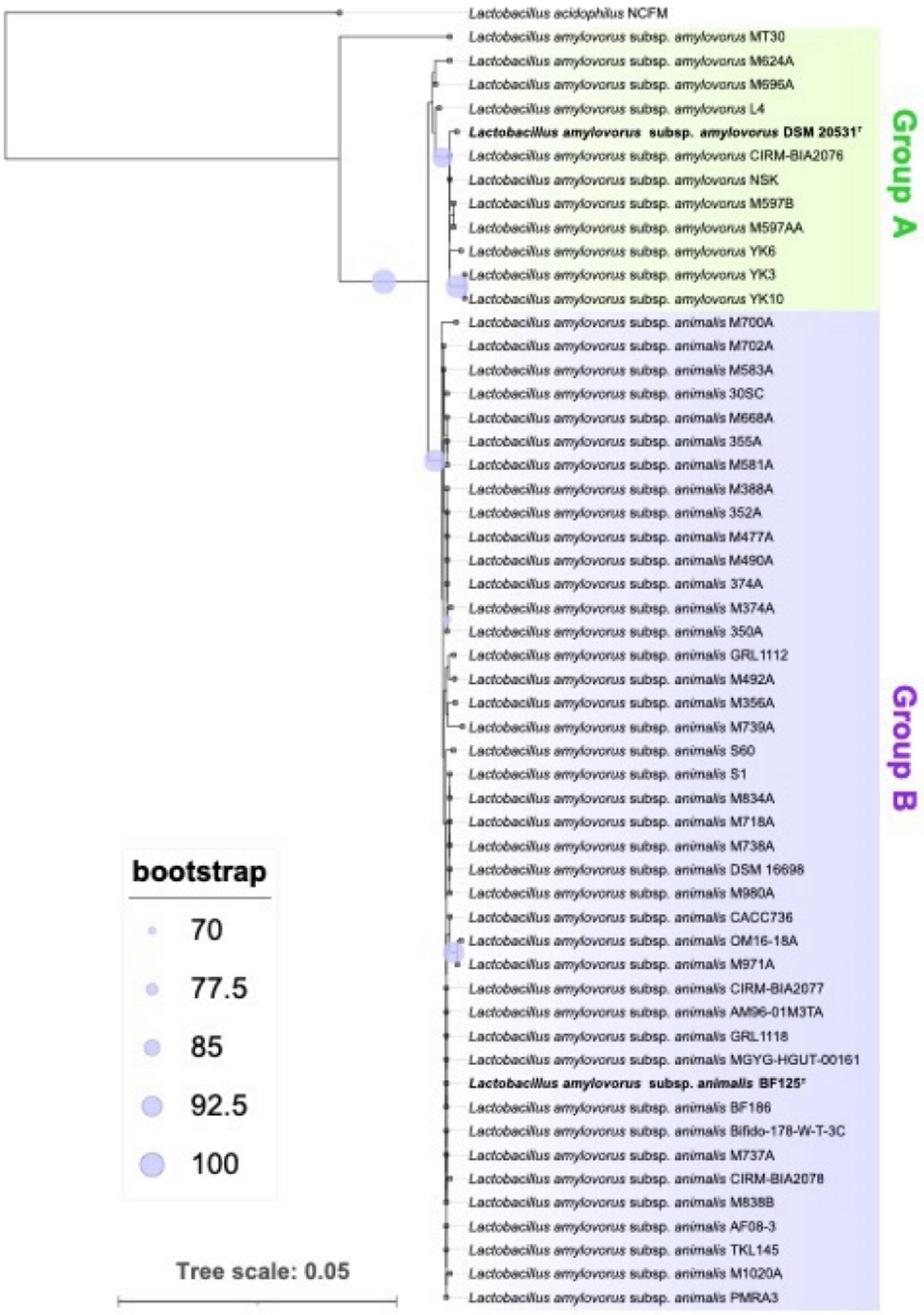

Supplementary Fig. S6. Neighbor-joining phylogenetic tree based on the nucleotide sequences of phospho-β-glucosidases of *L. amylovorus* (54 strains). The locus tags of the phospho-β-glucosidase genes used for this analysis are shown in Table S4. The nucleotide sequences were aligned with MAFFT (version 7.520) [27] in auto mode. The multiple sequence alignment was trimmed using trimAl (version 1.4.1) with the parameter "-gappyout" [28]. A tree based on Kimura's two-parameter model [29] was reconstructed using RapidNJ (version 2.3.2) [30]. Strains DSM 20531^T (representative of group A) and BF125^T (representative of group B) are highlighted in boldface type. The sequence of *Lactobacillus acidophilus* NCFM is used as an outgroup. Bootstrap values (1000 replicates) over 70% are indicated (blue circles). Bar, 0.05 substitutions per nucleotide sequences position.

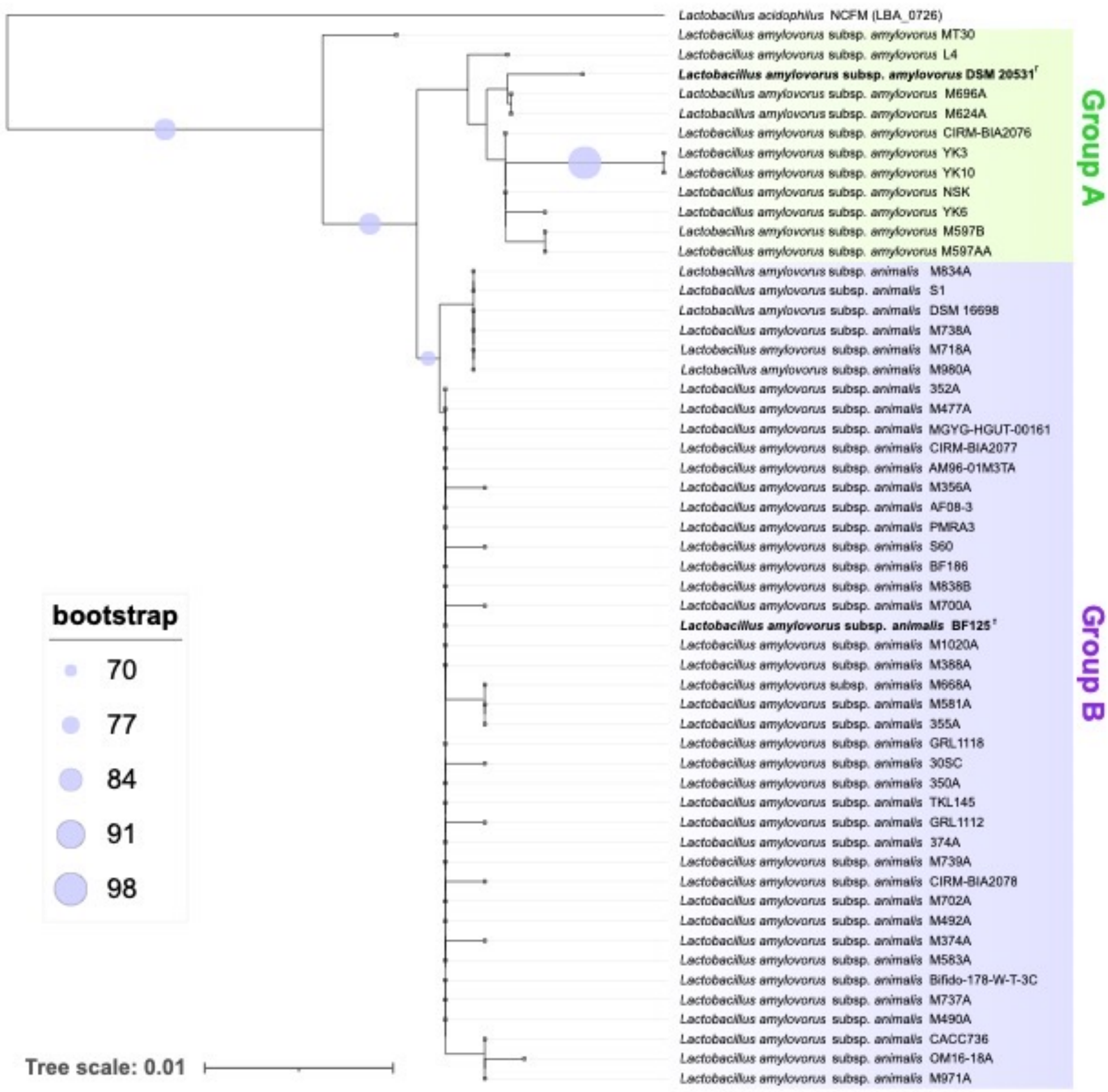

Supplementary Fig. S7. Neighbor-joining phylogenetic tree based on the amino acid sequences of phospho-β-glucosidases of *L. amylovorus* (54 strains). The predicted amino acid sequences are obtained from the corresponding locus tags (Table S4). The amino acid sequences were aligned with MAFFT (version 7.511) using the L-INS-i option, and the tree was constructed using the Jones–Taylar–Thornton model (Jones *et al.*, 1992) in the MAFFT online service (Katoh *et al.*, 2019). Strains DSM 20531^{T} (representative of group A) and BF125^{T} (representative of group B) are highlighted in boldface type. The sequence of *Lactobacillus acidophilus* NCFM is used as an outgroup. Bootstrap values (1000 replicates) over 70% are indicated (blue circles). Bar, 0.01 substitutions per amino acid sequence position.

References

Jones DT, Taylor WR, Thornton JM. The rapid generation of mutation data matrices from protein sequences. *Comput Appl Biosci* 1992;8(3):275-282. doi:10.1093/bioinformatics/8.3.275.

Katoh K, Rozewicki J, Yamada KD. MAFFT online service: multiple sequence alignment, interactive sequence choice and visualization. *Brief Bioinform* 2019;20(4):1160-1166. doi:10.1093/bib/bbx108

Supplementary Table S1. Statistics of the genome sequences obtained in this study (n=7).

Strains	Number of contigs	Total length (bp)	N50 (bp)	G+C ratio (%)	Gap ratio (%)	Depth coverage	Number of coding sequences	Completeness (%)	Contamination (%)	INSDC accession number
BF125^{T}	33	1,984,003	133,636	37.8	0.0	298x	2,018	99.03	0.16	BTFR01000001-BTFR01000033
BF186	77	1,969,667	91,131	37.8	0.0	306x	1,981	99.03	0.16	BTFQ01000001–BTFQ01000077
TKL145	72	2,002,886	65,994	38.0	0.0	354x	2,046	98.92	0.81	BAAAAK010000001–BAAAAK010000072
YK3	55	1,859,039	62,523	38.0	0.0	624x	1,903	98.92	0.0	BTFS01000001–BTFS01000055
YK6	32	1,885,203	117,657	38.0	0.0	617x	1,911	97.63	0.16	BTFT01000001–BTFT01000032
YK10	72	1,835,711	54,982	38.0	0.0	536x	1,855	98.92	0.0	BTFU01000001–BTFU01000072
NSK	72	1,973,634	56,116	37.9	0.0	302x	1,980	98.92	0.16	BAAAAG010000001–BAAAAG010000072

Supplementary Table S2. Reference *Lactobacillus amylovorus* genomes (n = 47) used for core genome analysis and phylogenomic tree construction

Species	Assembly accession number	Strain	Isolation source
L. amylovorus	GCF_000182855.2	GRL1112	Feces, pig
L. amylovorus	GCF_000191545.1	30SC	Intestines, swine
L. amylovorus	GCF_000194115.1	GRL1118	Ileum, porcine
L. amylovorus	GCF_001437365.1	DSM 16698	Feces, piglet intestine
L. amylovorus	GCF_002706375.1	DSM 20531^T	Cattle waste-corn fermentation
L. amylovorus	GCF_003465905.1	AF08-3	Feces, homo sapiens
L. amylovorus	GCF_005049155.1	S60	Nasopharynx, bovine
L. amylovorus	GCF_006384175.1	PMRA3	Feces, pig
L. amylovorus	GCF_012843555.1	Bifido-178-WT-3C	Feces, pig (3 month old, wild type)
L. amylovorus	GCF_020149995.1	MT30	Brewing environment of Maotai-flavor liquor
L. amylovorus	GCF_022642685.1	L4	Rectum, bos taurus
L. amylovorus	GCF_023523445.1	S1	Small intestine, pig
L. amylovorus	GCF_025194565.1	CIRM-BIA2076	unknown
L. amylovorus	GCF_025194575.1	CIRM-BIA2077	Anterior small intestinal contents, hog
L. amylovorus	GCF_025194605.1	CIRM-BIA2078	Small intestinal contents, hog
L. amylovorus	GCF_025449355.1	CACC736	Feces, pig
L. amylovorus	GCF_027691405.1	OM16-18A	Fecal materials, homo sapiens
L. amylovorus	GCF_027697865.1	AM96-01M3TA	Fecal materials, homo sapiens
L. amylovorus	GCF_027977705.1	M597AA	Intestinal contents, pig/wild boar
L. amylovorus	GCF_028308325.1	M1020A	Intestinal contents, pig/domestic pig
L. amylovorus	GCF_028308365.1	M971A	Intestinal contents, pig/domestic pig
L. amylovorus	GCF_028308375.1	M980A	Intestinal contents, pig/domestic pig
L. amylovorus	GCF_028308525.1	M838B	Intestinal contents, pig/domestic pig
L. amylovorus	GCF_028308535.1	M834A	Intestinal contents, pig/domestic pig
L. amylovorus	GCF_028308545.1	M739A	Intestinal contents, pig/domestic pig
L. amylovorus	GCF_028308585.1	M738A	Intestinal contents, pig/domestic pig
L. amylovorus	GCF_028308685.1	M737A	Intestinal contents, pig/domestic pig
L. amylovorus	GCF_028308695.1	M718A	Intestinal contents, pig/domestic pig
L. amylovorus	GCF_028308725.1	M668A	Intestinal contents, pig/wild boar
L. amylovorus	GCF_028308735.1	M702A	Intestinal contents, pig/wild boar
L. amylovorus	GCF_028308755.1	M700A	Intestinal contents, pig/wild boar
L. amylovorus	GCF_028308825.1	M696A	Intestinal contents, pig/wild boar
L. amylovorus	GCF_028308865.1	M624A	Intestinal contents, pig/wild boar
L. amylovorus	GCF_028308885.1	M597B	Intestinal contents, pig/wild boar
L. amylovorus	GCF_028308895.1	M583A	Intestinal contents, pig/wild boar
L. amylovorus	GCF_028308925.1	M581A	Intestinal contents, pig/wild boar
L. amylovorus	GCF_028308945.1	M492A	Intestinal contents, pig/wild boar
L. amylovorus	GCF_028308955.1	M490A	Intestinal contents, pig/wild boar
L. amylovorus	GCF_028308985.1	M374A	Intestinal contents, pig/wild boar
L. amylovorus	GCF_028308995.1	M388A	Intestinal contents, pig/wild boar
L. amylovorus	GCF_028309025.1	M477A	Intestinal contents, pig/wild boar
L. amylovorus	GCF_028309045.1	355A	Intestinal contents, pig/wild boar
L. amylovorus	GCF_028309055.1	M356A	Intestinal contents, pig/wild boar
L. amylovorus	GCF_028309085.1	374A	Intestinal contents, pig/wild boar
L. amylovorus	GCF_028309095.1	350A	Intestinal contents, pig/wild boar
L. amylovorus	GCF_028309125.1	352A	Intestinal contents, pig/wild boar
L. amylovorus	GCF_902363955.1	MGYG-HGUT-00161	Gut, human

Supplementary Table S3. Fatty acid compositions of *Lactobacillus amylovorus* subsp. *animalis* subsp. nov. and its closest phylogenetic relatives

Strains: 1, DSM 20531^T; 2, YK10; 3, NSK; 4, BF125^T; 5, DSM 16698. Each value shown is expressed as a percentage of the total fatty acids. tr, trace (<0.5 %); -, not detected; DMA, dimethylacetal; ECL, equivalent chain-length. All tested fatty acid profiles were determined under identical conditions in the present study.

**Fatty acid**		**1**	**2**	**3**	**4**	**5**
Saturated:						
	$C_{10:0}$	tr	tr	tr	tr	0.7
	$C_{12:0}$	2.3	tr	tr	tr	tr
	$C_{14:0}$	**17.1**	tr	4.5	tr	tr
	$C_{16:0}$	**39.8**	**24.0**	**53.3**	**22.7**	**23.8**
	$C_{16:0}$ 3OH	tr	-	tr	-	-
	$C_{18:0}$	2.0	3.1	1.9	3.4	3.3
	$C_{18:0}$ 12OH	1.7	2.1	1.2	1.7	3.4
Unsaturated:						
	$C_{16:1}$ ω7*c*	1.3	2.6	1.0	2.6	3.2
	$C_{18:1}$ ω9*c*	**14.0**	**25.9**	**12.1**	**31.3**	**27.6**
	$C_{18:1}$ ω7*c* DMA	1.2	2.0	1.2	2.0	2.5
Cyclopropane acids:						
	$C_{17:0}$ cyclopropane	-	-	tr	-	-
	C_{19} cyclopropane 9,10	**11.2**	**24.0**	**16.9**	**16.7**	**18.1**
Summed features*:						
	5	tr	-	tr	-	-
	8	-	-	-	tr	-
	10	7.0	**12.4**	5.8	**15.0**	**12.9**
	12	0.5	1.0	tr	1.2	1.0
Unknown ECL 18.199		1.2	2.3	2.3	2.2	2.8

* Summed features are fatty acids that cannot be resolved reliably from another fatty acid using the chromatographic conditions chosen. The MIDI system groups these fatty acids together as one feature with a single percentage of the total. Summed feature 5: $C_{15:0}$ DMA/$C_{14:0}$ 3OH; summed feature 8: $C_{18:1}$ ω8*c*/$C_{17:2}$ at 16.801 ; summed feature 10: $C_{18:1}$ ω7*c*/ unknown 17.834 ; summed feature 12: unknown ECL 18.622/$C_{19:0}$ iso.

Supplementary Table S4. Locus tags of phospho-β-glucosidase genes used for phylogenetic analysis

Locus Tag	Assembly accession number	Species	Strain	Isolation source
LA2_RS03850	GCF_000182855.2	*L. amylovorus* subsp. *animalis*	GRL1112	Feces, pig
LAC30SC_RS03810	GCF_000191545.1	*L. amylovorus* subsp. *animalis*	30SC	Intestines, swine
LAB52_RS03720	GCF_000194115.1	*L. amylovorus* subsp. *animalis*	GRL1118	Ileum, porcine
IV44_RS00740	GCF_001437365.1	*L. amylovorus* subsp. *animalis*	DSM 16698	Feces, piglet intestine
LA20531_RS09315	GCF_002706375.1	*L. amylovorus* subsp. *amylovorus*	DSM 20531^T	Cattle waste-corn fermentation
DWV49_RS02845	GCF_003465905.1	*L. amylovorus* subsp. *animalis*	AF08-3	Feces, homo sapiens
FCF10_RS01200	GCF_005049155.1	*L. amylovorus* subsp. *animalis*	S60	Nasopharynx, bovine
DM298_RS07240	GCF_006384175.1	*L. amylovorus* subsp. *animalis*	PMRA3	Feces, pig
HF869_RS09225	GCF_012843555.1	*L. amylovorus* subsp. *animalis*	Bifido-178-WT-3C	Feces, pig (3 month old, wild type)
LA162_RS06680	GCF_020149995.1	*L. amylovorus* subsp. *amylovorus*	MT30	Brewing environment of Maotai-flavor liquor
G8B20_RS05900	GCF_022642685.1	*L. amylovorus* subsp. *amylovorus*	L4	Rectum, bos taurus
L0B74_RS07150	GCF_023523445.1	*L. amylovorus* subsp. *animalis*	S1	Small intestine, pig
EFR95_RS06400	GCF_025194565.1	*L. amylovorus* subsp. *amylovorus*	CIRM-BIA2076	unknown
EFS00_RS09965	GCF_025194575.1	*L. amylovorus* subsp. *animalis*	CIRM-BIA2077	Anterior small intestinal contents, hog
EFR91_RS02225	GCF_025194605.1	*L. amylovorus* subsp. *animalis*	CIRM-BIA2078	Small intestinal contents, hog
N6G93_RS03180	GCF_025449355.1	*L. amylovorus* subsp. *animalis*	CACC736	Feces, pig
PG087_RS00035	GCF_027691405.1	*L. amylovorus* subsp. *animalis*	OM16-18A	Fecal materials, homo sapiens
PG784_RS03520	GCF_027697865.1	*L. amylovorus* subsp. *animalis*	AM96-01M3TA	Fecal materials, homo sapiens
ODV19_RS03855	GCF_027977705.1	*L. amylovorus* subsp. *amylovorus*	M597AA	Intestinal contents, pig/wild boar
ODV25_RS04885	GCF_028308325.1	*L. amylovorus* subsp. *animalis*	M1020A	Intestinal contents, pig/domestic pig
ODV29_RS07960	GCF_028308365.1	*L. amylovorus* subsp. *animalis*	M971A	Intestinal contents, pig/domestic pig
ODV20_RS03035	GCF_028308375.1	*L. amylovorus* subsp. *animalis*	M980A	Intestinal contents, pig/domestic pig
ODV30_RS08185	GCF_028308525.1	*L. amylovorus* subsp. *animalis*	M838B	Intestinal contents, pig/domestic pig
ODV81_RS03885	GCF_028308535.1	*L. amylovorus* subsp. *animalis*	M834A	Intestinal contents, pig/domestic pig
ODV23_RS05140	GCF_028308545.1	*L. amylovorus* subsp. *animalis*	M739A	Intestinal contents, pig/domestic pig
ODV28_RS01180	GCF_028308585.1	*L. amylovorus* subsp. *animalis*	M738A	Intestinal contents, pig/domestic pig
ODV26_RS05700	GCF_028308685.1	*L. amylovorus* subsp. *animalis*	M737A	Intestinal contents, pig/domestic pig
ODV24_RS06100	GCF_028308695.1	*L. amylovorus* subsp. *animalis*	M718A	Intestinal contents, pig/domestic pig
ODV21_RS01300	GCF_028308725.1	*L. amylovorus* subsp. *animalis*	M668A	Intestinal contents, pig/wild boar
ODV82_RS02340	GCF_028308735.1	*L. amylovorus* subsp. *animalis*	M702A	Intestinal contents, pig/wild boar
ODV22_RS00320	GCF_028308755.1	*L. amylovorus* subsp. *animalis*	M700A	Intestinal contents, pig/wild boar
ODV17_RS01700	GCF_028308825.1	*L. amylovorus* subsp. *amylovorus*	M696A	Intestinal contents, pig/wild boar
ODV08_RS04440	GCF_028308865.1	*L. amylovorus* subsp. *amylovorus*	M624A	Intestinal contents, pig/wild boar
ODV14_RS03310	GCF_028308885.1	*L. amylovorus* subsp. *amylovorus*	M597B	Intestinal contents, pig/wild boar
ODV10_RS00340	GCF_028308895.1	*L. amylovorus* subsp. *animalis*	M583A	Intestinal contents, pig/wild boar
ODV16_RS00325	GCF_028308925.1	*L. amylovorus* subsp. *animalis*	M581A	Intestinal contents, pig/wild boar
ODV12_RS05355	GCF_028308945.1	*L. amylovorus* subsp. *animalis*	M492A	Intestinal contents, pig/wild boar
ODU72_RS05105	GCF_028308955.1	*L. amylovorus* subsp. *animalis*	M490A	Intestinal contents, pig/wild boar
ODV09_RS01300	GCF_028308985.1	*L. amylovorus* subsp. *animalis*	M374A	Intestinal contents, pig/wild boar
ODV11_RS00740	GCF_028308995.1	*L. amylovorus* subsp. *animalis*	M388A	Intestinal contents, pig/wild boar
ODV13_RS07970	GCF_028309025.1	*L. amylovorus* subsp. *animalis*	M477A	Intestinal contents, pig/wild boar
ODU82_RS04065	GCF_028309045.1	*L. amylovorus* subsp. *animalis*	355A	Intestinal contents, pig/wild boar
ODV15_RS06460	GCF_028309055.1	*L. amylovorus* subsp. *animalis*	M356A	Intestinal contents, pig/wild boar
ODU74_RS02080	GCF_028309085.1	*L. amylovorus* subsp. *animalis*	374A	Intestinal contents, pig/wild boar
ODU71_RS02800	GCF_028309095.1	*L. amylovorus* subsp. *animalis*	350A	Intestinal contents, pig/wild boar
ODU75_RS08045	GCF_028309125.1	*L. amylovorus* subsp. *animalis*	352A	Intestinal contents, pig/wild boar
FY235_RS02835	GCF_902363955.1	*L. amylovorus* subsp. *animalis*	MGYG-HGUT-00161	Gut, human
AAA895_RS03860	GCF_036245395.1	*L. amylovorus* subsp. *animalis*	BF125^T	Feces, bovine
AAA909_RS04850	GCF_036245375.1	*L. amylovorus* subsp. *animalis*	BF186	Feces, bovine
ABXG80_RS07925	GCF_040364785.1	*L. amylovorus* subsp. *amylovorus*	NSK	Steeping water of corn starch product plant
ABWU77_RS04940	GCF_040365475.1	*L. amylovorus* subsp. *animalis*	TKL145	Feces, porcine
AAA905_RS07920	GCF_036245415.1	*L. amylovorus* subsp. *amylovorus*	YK3	Spent mushroom substrates
AAA883_RS00595	GCF_036245435.1	*L. amylovorus* subsp. *amylovorus*	YK6	Spent mushroom substrates
AAA891_RS07600	GCF_036245455.1	*L. amylovorus* subsp. *amylovorus*	YK10	Spent mushroom substrates
LBA_0726	GCF_000011985.1	*L. acidophilus*	NCFM	Feces, infant
